# Potential of Pan-Tuberculosis Treatment to Drive Emergence of Novel Resistance

**DOI:** 10.3201/eid3008.240541

**Published:** 2024-08

**Authors:** C. Finn McQuaid, Theresa S. Ryckman, Nicolas A. Menzies, Richard G. White, Ted Cohen, Emily A. Kendall

**Affiliations:** London School of Hygiene and Tropical Medicine, London, UK (C.F. McQuaid, R.G. White);; Johns Hopkins University School of Medicine, Baltimore, Maryland, USA (T.S. Ryckman, E.A. Kendall);; Harvard T.H. Chan School of Public Health, Boston, Massachusetts, USA (N.A. Menzies);; Yale School of Public Health, New Haven, Connecticut, USA (T. Cohen)

**Keywords:** tuberculosis and other mycobacteria, antimicrobial resistance, bacteria, TB

## Abstract

New tuberculosis (TB) drugs with little existing antimicrobial resistance enable a pan-TB treatment regimen, intended for universal use without prior drug-susceptibility testing. However, widespread use of such a regimen could contribute to an increasing prevalence of antimicrobial resistance, potentially rendering the pan-TB regimen ineffective or driving clinically problematic patterns of resistance. We developed a model of multiple sequential TB patient cohorts to compare treatment outcomes between continued use of current standards of care (guided by rifampin-susceptibility testing) and a hypothetical pan-TB approach. A pan-TB regimen that met current target profiles was likely to initially outperform the standard of care; however, a rising prevalence of transmitted resistance to component drugs could make underperformance likely among subsequent cohorts. Although the pan-TB approach led to an increased prevalence of resistance to novel drugs, it was unlikely to cause accumulation of concurrent resistance to novel drugs and current first-line drugs.

Rifampin-resistant tuberculosis (RR-TB) is a key contributor to global antimicrobial resistance; >400,000 persons had RR-TB in 2020, nearly 40% of whom probably died as a result (*1*). More than 57% of those who have RR-TB disease are not enrolled in RR-TB treatment, including those who are not treated for tuberculosis (TB) at all and those who are inappropriately given treatments for rifampin-susceptible TB (RS-TB) (*1*). For those who are enrolled in RR-TB treatment, recent advances, including 6-month oral regimens (*2*), have improved tolerability and increased cure rates to >80%, but the need to identify drug resistance and direct patients with RR-TB down a second-line treatment pathway still complicates the diagnosis and treatment of TB.

Although tailored treatment remains a key pillar in preventing antimicrobial-resistance in general, a lack of access to rapid drug-susceptibility testing (DST) for TB represents a critical issue in reducing inappropriate treatment, in which drugs for TB treatment are almost solely used for TB. The growing availability of novel drugs and drug candidates with low or no resistance (*3*,*4*) has enabled a pan-TB treatment approach, offering 1 universal treatment regimen and no requirement for DST before initiating treatment. The World Health Organization (WHO) target profile for a pan-TB regimen also calls for short duration and improved tolerability and forgiveness (i.e., ability to withstand nonadherence without negative consequences) compared with the current standard of care (*5*). Such a regimen has the potential to remove barriers to initiation of appropriate treatment, improve treatment outcomes, and be effective and cost-effective (*6*). Although the regimen could be highly effective, the pan-TB approach requires that resistance to its component drugs is rare.

One concern about such a regimen is that widespread use of new drugs could exert a selective pressure that favors increases in resistance. Testing for both phenotypic and genotypic resistance to novel drugs remains limited and, because of technical difficulties, is likely to remain so in the near future (*7*). In the context of emerging resistance to a pan-TB regimen’s component drugs, continued use of the regimen without routine susceptibility testing could lead to resistance to multiple drugs in the regimen and poor clinical outcomes. In addition, use of current first-line drugs such as rifampin to retreat those with resistance to the pan-TB regimen could lead to the development of TB strains simultaneously resistant to both pan-TB drugs and current first-line drugs, leaving limited treatment options for affected persons.

Those resistance-related risks warrant particularly careful consideration given that leading candidate regimens in development for the pan-TB indication share newer drugs such as bedaquiline and pretomanid with the regimens currently recommended for treating RR-TB (clinical trial nos. NCT05971602 [https://www.clinicaltrials.gov/study/NCT05971602] and NCT06114628 [https://www.clinicaltrials.gov/study/NCT06114628]) (*8*–*11*). Although most TB, including most RR-TB, remains susceptible to these new drugs, resistance has emerged quickly in some patient populations for whom they have been used. For example, in South Africa, an early adopter of bedaquiline for RR-TB treatment, as much as 8% of the 2017 RR-TB cohort also had bedaquiline resistance (*12*). Those data suggest that resistance to some pan-TB regimen components might be prevalent among patients with RR-TB by the time a pan-TB regimen becomes available and that similar emergence could occur among RS-TB if pan-TB regimens are not designed to guard against emergent resistance.

Evidence is limited on the pace of emerging resistance or its effects on treatment outcomes, but an urgent need exists to anticipate pathways by which resistance could emerge. We used a modeling approach to explore the circumstances under which use of a pan-TB regimen could contribute to increasing resistance prevalence to new and existing drugs, posing resistance-related risks that could compromise the overall health benefit of a pan-TB strategy.

## Methods

**Figure 1:**
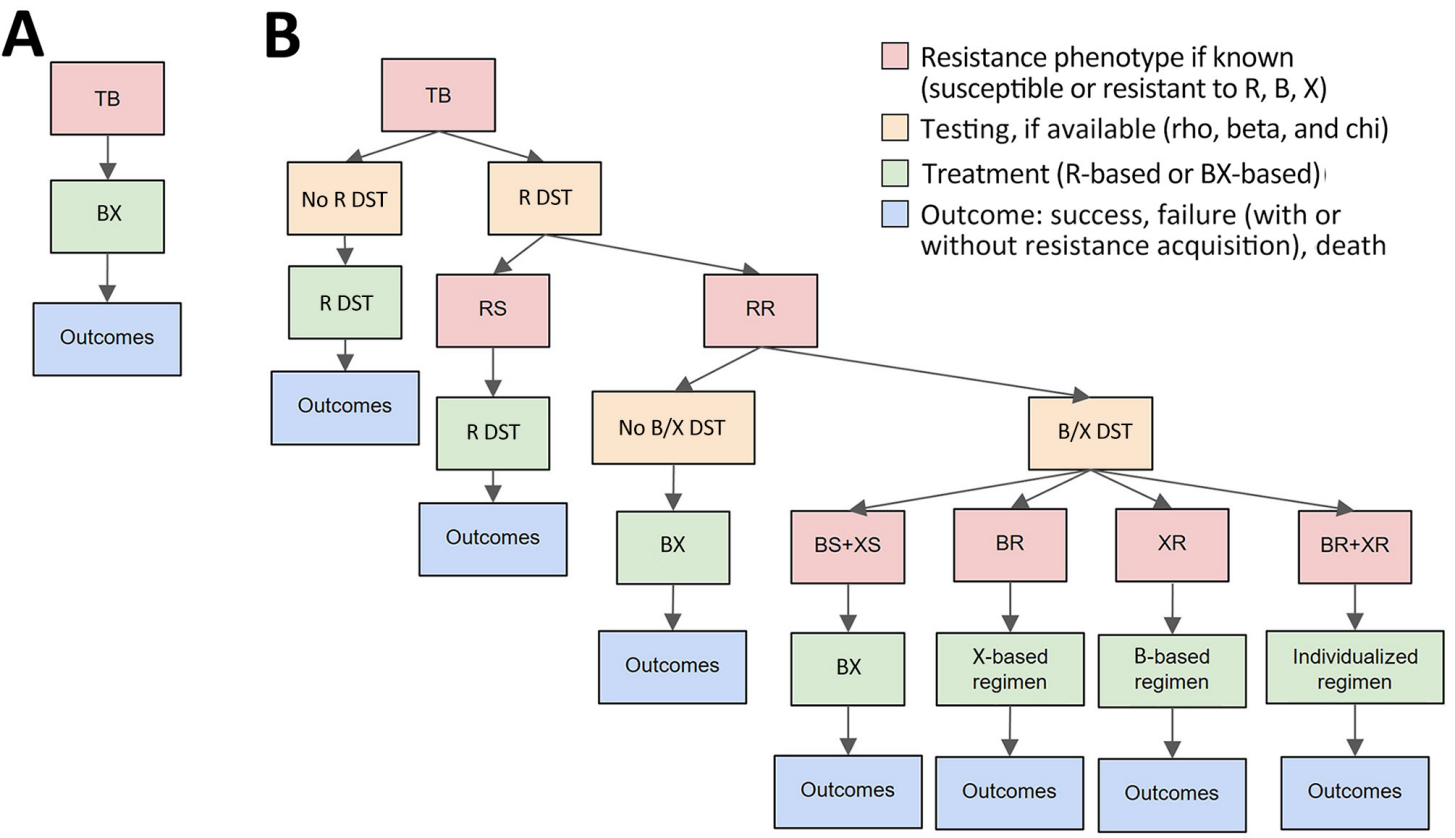
Treatment pathways for new TB patients in study of potential of pan-TB treatment to drive emergence of novel resistance, comparing a pan-TB treatment scenario (A) with the standard-of-care scenario (B). Retreatment pathways are shown in Appendix Figure 1. BR-TB, diarylquinoline-resistant TB; RR-TB, rifampin-resistant TB; RS-TB, rifampin-susceptible TB; TB, tuberculosis; XR-TB, TB resistant to additional novel drug X; R DST, rifampin drug-susceptibility testing; B DST, diarylquinoline susceptibility testing; X DST, other novel drug (or drugs) susceptibility testing.

We developed a cohort model to evaluate clinical and drug-resistance outcomes over multiple successive cohorts of patients (Appendix Table 1) (Figure 1). We followed each cohort from the time of initial TB diagnosis, comparing a pan-TB strategy with current standards of care. 

### Modeling of Drugs, Regimens, and Drug Resistance

We explicitly modeled 3 drug classes as components of treatment regimens and drug-susceptibility phenotypes: rifamycins including rifampin (R), diarylquinolines including bedaquiline (B), and an additional unspecified novel drug (X) that is a component of RR-TB and pan-TB regimens. X was modeled on bedaquiline in its propensity to develop drug-resistance and the effects of that drug resistance on treatment outcome. The standard-of-care regimen for treatment of RS-TB is denoted R but implicitly includes additional drugs such as isoniazid, and the B- and X- containing regimen (BX) implicitly includes >1 additional novel drugs.

Phenotypes resistant to R, B, and X are shown as RR-TB, BR-TB, and XR-TB, respectively. Those phenotypic categories enable resistance to additional drugs whose resistance was not explicitly modeled; for example, RR-TB is usually isoniazid-resistant and may be fluoroquinolone-resistant, and XR-TB may be resistant to >1 components of the pan-TB regimen. We modeled resistance to each drug in simplified binary fashion, corresponding to accepted breakpoints for phenotypic resistance to rifampin and bedaquiline. We defined concurrent resistance both to R and to either B, X, or both B and X as complex resistance in our model. We combined DST coverage and sensitivity to detect RR, BR, and XR phenotypes into probability-of-resistance-detection parameters.

### Initial Treatment Pathways

In the standard-of-care scenario, R was used to treat RS-TB. We assumed current levels of DST coverage for rifampin. BX was used for treatment of RR-TB, reflecting current WHO guidelines recommending BPaL(M) (*13*), and we assumed that future improvements to the efficacy or safety of this regimen’s component classes would be incorporated into future RR-TB regimens. We assumed that only a fraction of patients would undergo DST for B and X before RR-TB treatment, estimated based on current fluoroquinolone DST coverage. For patients with detected resistance to rifamycins and >1 novel drugs (B or X), we assumed that an individualized second-line regimen would be constructed, with inclusion of either B or X (i.e., an X-based or B-based individualized regimen) if susceptibility to 1 of these drugs was retained and a conventional second-line regimen otherwise.

In the pan-TB scenario, we assumed use of the BX regimen for all new TB patients. In addition, no DST was conducted for any drugs before initial treatment.

### Treatment Outcomes

We modeled 4 possible outcomes of TB treatment: durable cure, non-cure (i.e., treatment failure or relapse) without acquisition of new resistance, non-cure with acquisition of new resistance to some component of the treatment regimen, and death. Probabilities of cure accounted for nonadherence, the possibility of early treatment discontinuation, and the effects of any preexisting resistance (Appendix Table 2); we separately estimated the likely outcome for each possible combination of regimen and pretreatment resistance phenotype (Appendix Table 3). Differences between regimens in terms of efficacy, duration of treatment, tolerability, and forgiveness correspond to a pan-TB target product profile developed by WHO (*5*), and we implicitly represented these factors as determinants of the proportion of patients durably cured on the basis of a previous study (*14*). For example, we implicitly represented the effect of poor treatment adherence attributable to low tolerability through a decreased probability of durable cure, dependent on regimen forgiveness. We assumed the same programmatic support across all regimens and did not explicitly consider regimen cost (including monitoring for side effects and other programmatic support) or ease of access to drugs, although those factors might be expected to have a differential effect on regimen use. We did not model rare acquisition of resistance to multiple regimen components during 1 treatment course.

### Retreatment Pathways

For patients who did not recover or who relapsed after a first round of treatment, the selection of retreatment regimens took into account the previous treatment regimen, any previously known drug resistance and, potentially, the results of additional or repeat DST (Appendix Figure 1). In the standard-of-care scenario, the mapping between known drug resistance and selected regimen was the same for retreatment as for initial treatment, but DST coverage for R (among patients not already known to have RR-TB) and for B and X (among patients with RR-TB) was higher in retreatment. In the pan-TB scenario, R was used as a retreatment regimen for patients with confirmed rifampin susceptibility, but BX continued to be the default regimen in retreatment for patients with no DST results. DST coverage for retreatment patients in the pan-TB scenario was the same as for new patients in the standard-of-care scenario. We also performed a sensitivity analysis where DST was performed at these levels for R but not for B or X and where DST coverage for R was 100%.

### Time Approximation

To simulate the accumulation of resistance and its effect on outcomes over time, we extended the cohort model over multiple treatment cohorts representing successive generations of transmitted TB. Our representation captured the number of generations of transmission but ignored the (large variation in) calendar time per generation. In estimating how transmission from earlier cohorts contributed to a future cohort of new TB patients, we assumed that cohort sizes remained the same over time (i.e., an effective reproduction number of 1 in all scenarios). The distribution of resistance in each cohort (but not the absolute size of the cohort) assumed that patients who had been unsuccessfully treated in previous cohorts had generated on average as much transmission after their initial diagnosis as before. Therefore, the initial drug-resistance composition of a given treatment cohort was a weighted average of the immediately preceding cohort at 3 different timepoints (at the start of treatment, after initial treatment, and after retreatment) weighted by the proportion of the cohort still with TB at each of those timepoints. We modeled 10 generations of new treatment cohorts.

Although our results can be loosely translated into calendar time (assuming, for example, that 1 cohort generation of transmission corresponds to a serial interval of 1–2 years on average [*15*]), our estimation of timescales does not take into account the distributions of times to secondary case generation or to diagnosis, both of which can be highly varied and setting-dependent for TB (*16*–*18*). Overinterpretation of the generation time is inadvisable.

We assumed that 4% of cases in the first cohort were RR-TB (*1*), of which 2% were also resistant to B, on the basis of recent clinical data on bedaquiline resistance (*12*,*19*–*21*). Simultaneously, 0.2% of RS-TB cases had resistance to B, also assumed on the basis of clinical trial and surveillance data for bedaquiline (*22*,*23*). We assumed that initial prevalence of resistance to X was zero. A sensitivity analysis considered higher baseline prevalence of BR (by the same relative factor among both RS-TB and RR-TB cases) to account for the possible accumulation of resistance between the present day and when pan-TB may become available in the future.

### Parameter Uncertainty

Many of our parameter values had little data to inform them. As such, we selected wide uncertainty ranges on the basis of the range of estimates available in the literature. We assumed β distributions for all parameter values except the risk ratio describing the effect of existing resistance on further resistance acquisition (Appendix Table 1), for which we assumed a uniform distribution because of the extremely high uncertainty and a desire to explore extreme values. To propagate uncertainty through the analysis, we created 1,000 parameter sets simultaneously, independently sampling from the distributions of all uncertain parameters, and we reestimated the model with each of these parameter sets. We explored parameter extremes through 1- and 2-way sensitivity analyses.

## Results

### Model Approach

**Figure 2:**
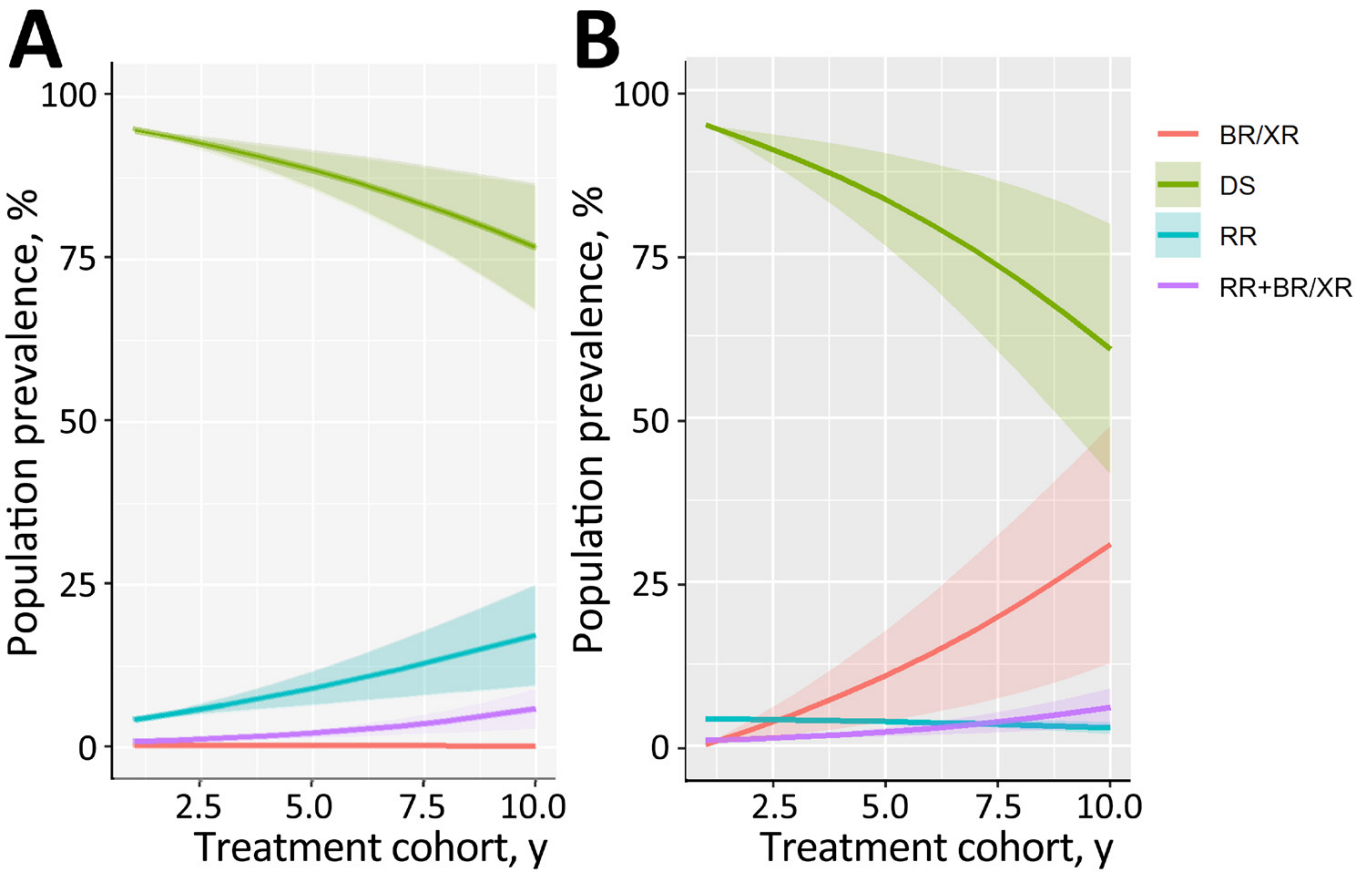
Prevalence of resistance phenotypes over multiple cohorts of TB treatment in study of potential of pan-TB treatment to drive emergence of novel resistance for standard-of-care (A) and pan-TB (B) treatment scenarios. Shaded areas indicate 95% CIs. x-axis indicates scale indicates treatment generations. BR/XR, TB resistant to a diarylquinoline or novel drug X or both only; DS, drug-susceptible TB; RR, rifampin-resistant TB; RR+BR/XR, TB resistant both to rifampin and to either a diarylquinoline, novel drug X, or both; TB, tuberculosis.

Over multiple cohorts of patients in both scenarios, the proportion of TB that was resistant to >1 modeled drug was projected to increase (i.e., the proportion of cases that was DS was projected to decrease) (Figure 2). The change was estimated to be slightly faster in the pan-TB scenario, such that by the 10th generation, only 61.0% (95% CI 42.3%–79.7%) of patients had no drug resistance under the pan-TB scenario, compared with 76.7% (95% CI 67.3%–86.0%) under the standard of care. However, those are proportion-based results and do not show changes in absolute TB incidence or drug-resistant TB incidence that may result from improved regimens.

The drug resistance that initially emerged under the pan-TB scenario was primarily B or X mono-resistance (mirroring the continued selection of R resistance in the standard-of-care scenario), but over time, with DST-free use of the pan-TB regimen, simultaneous resistance to both B and X became more common (30.8% [95% CI 12.6%–48.9%] of all TB cases after 10 cohorts of treatment) (Figure 2). We examined the routes by which patients with different resistance phenotypes arrive at their final treatment outcomes (Appendix Figure 2). 

### Performance of Pan-TB versus Standard of Care

**Figure 3:**
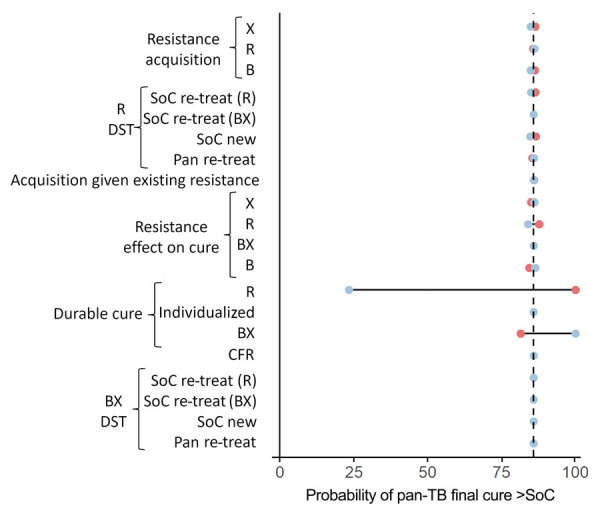
Univariate sensitivity analysis for initial TB treatment regimen comparison in study of potential of pan-TB treatment to drive emergence of novel resistance. Parameter sets are sampled with 1 parameter fixed at the extremes of its 95% CI. The outcome is the proportion of samples that result in more patients durably cured in the pan-TB scenario than the SoC scenario, within the first cohort of patients treated, and at current prevalence of resistance. Blue circles indicate use of upper bound of the parameter’s 95% CI; red circles indicate the lower bound. B, diarylquinolines; BX, diarylquinoline- and novel drug X–containing regimen; CFR, case-fatality ratio; DST, drug-susceptibility testing; R, rifamycins; re-treat, patients with previously treated TB; SoC, standard of care; X, additional novel drug X.

For a single cohort of patients, when sampling our uncertainty distributions for all parameters (including initial prevalences of resistance) simultaneously, pan-TB was highly (85.9%) likely to result in more patients cured than the standard of care (Figure 3). In univariate analysis (fixing 1 parameter at an extreme of its uncertainty range while varying all others), the probability of standard of care outperforming pan-TB was observed to depend most heavily on the relative effectiveness of the pan-TB regimen compared with the standard of care in achieving durable cure among patients with DS-TB. The outperformance of the pan-TB regimen compared with the standard of care, in an initial cohort of patients, was robust to variation in any single resistance-related parameter and to simultaneous variation in the prevalence of both R and B resistance (Appendix Figure 3); however, assuming a higher prevalence of B resistance increased the sensitivity to other uncertain parameters (Appendix Figure 4).

### Pan-TB Viability over Time

**Figure 4:**
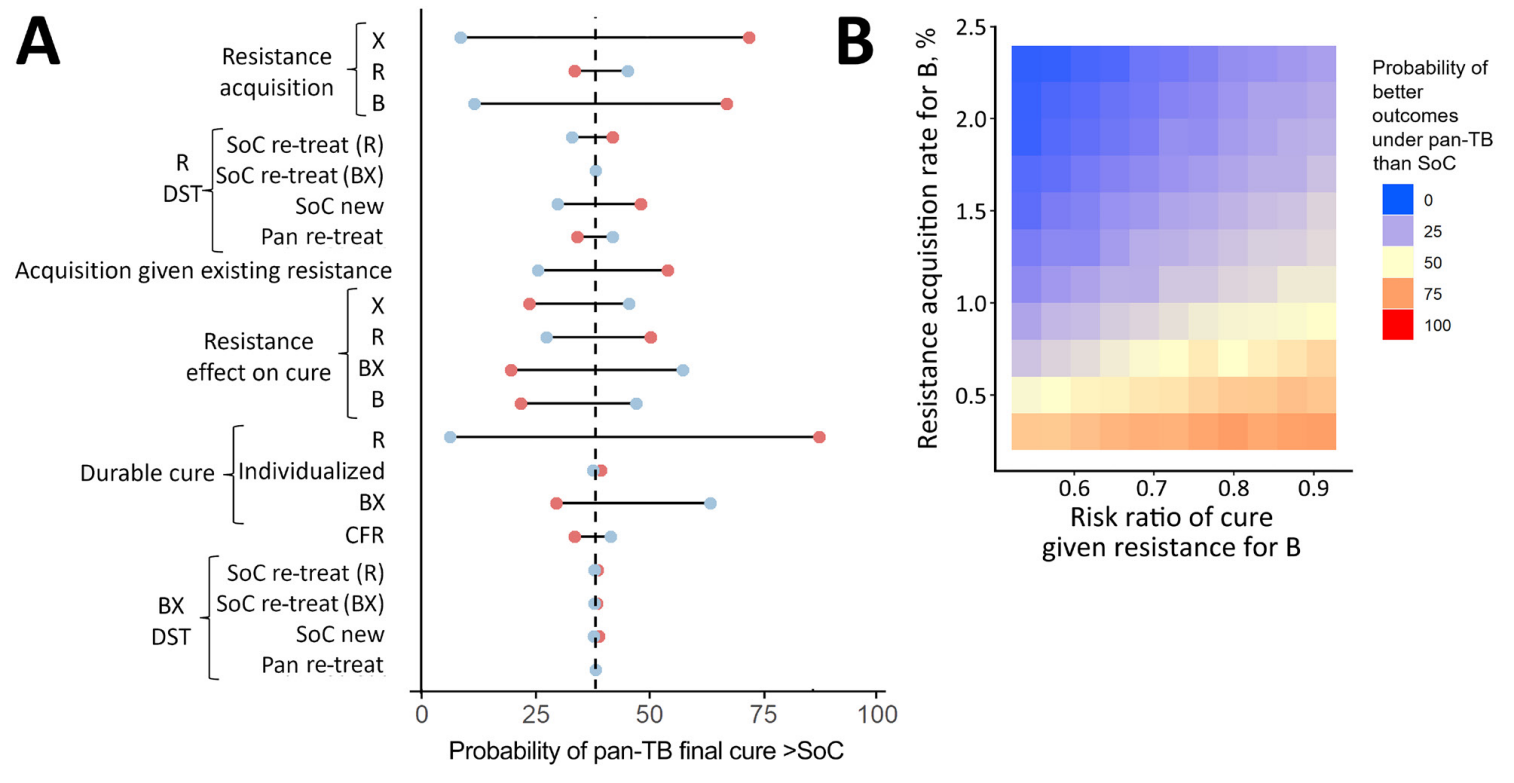
Sensitivity analysis for TB treatment regimen comparison after use in multiple patient cohorts in study of potential of pan-TB treatment to drive emergence of novel resistance. Comparison shows an outcome of proportion of patients durably cured in the 10th cohort when using either the pan-TB or the SoC approach for 10 cohorts. A) Univariate sensitivity analysis, sampling parameter sets with 1 parameter fixed at an extreme of its 95% CI, where blue circles indicate high parameter values and red circles low parameter values. B) Multivariate sensitivity analysis varying 2 resistance-related parameters simultaneously, where red indicates when pan-TB TB regimen performs better and blue when SoC regimen performs better. B, diarylquinolines; BX, diarylquinoline- and novel drug X–containing regimen; CFR, case-fatality ratio; DST, drug susceptibility testing; R, rifamycins; re-treat, those with previously treated TB; SoC, standard of care; X, additional novel drug X.

In addition to modeling the treatment outcomes of a single cohort with current levels of initial drug resistance (Figure 3), we assessed how the effects of uncertain model parameters can compound over multiple generations of transmission and treatment by comparing multiple generations of the standard of care to multiple generations of the pan-TB approach for each sampled set of parameters (Figure 4). We estimated that the probability of pan-TB outperforming the standard of care in a patient cohort would drop to 38% within 10 generations (Figure 4, panel A) compared with 86% in the initial patient cohort (Figure 3).

In sensitivity analyses that propagated uncertainty through multiple patient cohorts, the comparison between regimen outcomes in later cohorts remained sensitive to the regimens’ relative effectiveness for DS-TB, but resistance-related parameters also increased in importance. The probability that the pan-TB regimen remained superior to the standard of care after 10 generations could vary from 11% to 68% as a result of varying the per-treatment risk for acquired B resistance and from 22% to 48% when varying the effects of that B resistance on efficacy. The durability of the pan-TB approach was even more certain to be short when these 2 parameters were varied simultaneously (Figure 4, panel B), when the corresponding parameters for both B and novel drug X were varied simultaneously (Appendix Figure 5), or when the rate of acquisition of resistance to novel drugs was increased (by extending the upper bound of the 95% CIs to 8%, as has been reported among RR-TB cohorts treated with bedaquiline, for example [*13*,*24*], and under programmatic conditions for earlier regimens [*25*]) (Appendix Figure 6).

### Emergence of Complex Resistance

**Figure 5:**
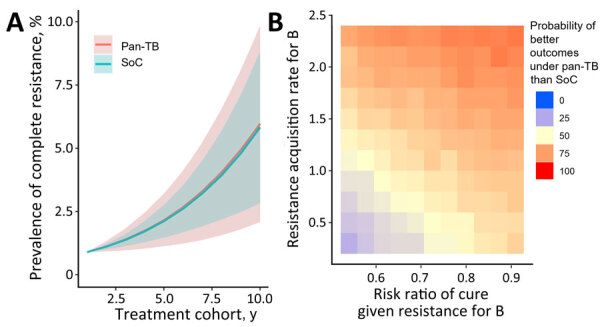
Prevalence of complex resistance to both R and a pan-TB treatment regimen component (B, X, or both) resulting from the pan-TB compared with standard-of-care scenario in study of potential of pan-TB treatment to drive emergence of novel resistance. A) Prevalence over treatment generations; B) probability that the pan-TB scenario leads to higher prevalence of complex resistance as a proportion of all TB after 10 cohorts. Red indicates when pan-TB regimen performs better (<50% probability of higher prevalence of complex resistance) and blue when SoC regimen performs better. B, diarylquinolines; SoC, standard of care; TB, tuberculosis.

The prevalence of concurrent, complex resistance both to R and to a novel drug (B, X, or both) increased at a similarly slow pace in both scenarios. The prevalence of complex resistance reached 6.0% (95% 2.3%–9.8%) of all TB in the 10th treatment generation of the pan-TB scenario and 5.9% (95% CI 2.9%–9.0%) in the 10th generation under the standard of care (Figure 5, panel A). These trends in complex resistance were largely unaffected by the availability of R DST and B or X DST for retreatment patients in the pan-TB scenario (Appendix Figure 7). The pan-TB scenario led to a higher prevalence of complex resistance than the standard of care when the acquisition rate of resistance to novel drugs was low (such that much of the B resistance reflected selection among RR-TB before introduction of the pan-TB regimen) and the effect of that resistance on cure was high (Figure 5, panel B; Appendix Figure 8).

## Discussion

Assuming that a pan-TB regimen was similarly efficacious against pan-susceptible TB as the current rifampin-susceptible TB regimen (leading to noninferiority under optimal trial conditions) and also had a shorter duration and improved adherence (resulting in high effectiveness under real-world conditions), we found that the pan-TB approach was very likely to outperform the standard of care when initially introduced, even with high levels of bedaquiline resistance in the population. As resistance to the pan-TB regimen accumulated (including resistance to multiple component novel drugs), the probability of outperformance declined rapidly. Still, the pan-TB approach was unlikely to cause accumulation of concurrent resistance to both novel and current drugs.

Our results affirm that introduction of a pan-TB regimen meeting current regimen-development targets is highly likely to initially improve populationwide treatment outcomes, primarily as a result of improved outcomes for patients with DS-TB. However, our results also demonstrate the likelihood that, after several cycles of transmission, continued use of a single pan-TB regimen will no longer be viable because of emerging resistance, including resistance to multiple components of the pan-TB regimen. Some combination of regimen improvements and DST reintroduction would be needed to maintain the improved health outcomes that the pan-TB regimen had initially offered. Given a pipeline of more potent diarylquinolines and novel drug classes (*4*), updating a pan-TB regimen to counteract this decline might be possible; however, further work is needed to compare the timescales of this emergence of resistance to those of regimen development and TB elimination and to understand the most effective responses to emerging resistance and their optimal timing.

Another potential concern about using novel drugs such as bedaquiline in all patients is that the broader population scale of acquired resistance might leave many patients without effective treatment options. We did not identify any clinical pathways by which this scenario was likely to occur. In our model, RR-TB was treated similarly in both scenarios (dependent on DST coverage) and showed high enough rates of durable cure to cause preexisting RR-TB to decrease over time in the pan-TB scenario. Meanwhile, although we modeled retreatment with a rifamycin-based regimen for a substantial proportion of RS-TB cases that were not cured with initial pan-TB regimen treatment (some of which had resistance to B, X, or both), we projected similar levels of complex resistance with this approach as with continued use of a rifamycin-based first-line regimen followed by use of novel drugs after selection of rifampin resistance. This finding suggests that, even as resistance accumulated to components of a pan-TB regimen, most patients for whom the pan-TB regimen was ineffective would (once identified by DST) have existing rifamycin-based regimens as back-ups.

In reality, many of the characteristics of future pan-TB regimens are unknown. Although the pan-TB target regimen profile seeks to improve regimen duration, tolerability, and pharmacologic forgiveness, the effects of those improvements on adherence and ultimately effectiveness may be unpredictable and setting-dependent. Our model suggests that if the difference in effectiveness is small between a pan-TB regimen and the rifamycin-based alternative for treating RS-TB, then the health advantages of the pan-TB regimen will be smaller and contingent on maintaining a low prevalence of resistance to drugs in the pan-TB regimen. Moreover, even for a pan-TB regimen much more effective than the standard of care, unfavorable resistance-related regimen characteristics could eventually threaten that effectiveness. For a pan-TB regimen to continue to achieve high rates of cure in the long term, it needs to be constructed to guard against acquisition of resistance to all of its components or to ensure that regimen efficacy remains high in the presence of any forms of resistance that are likely to emerge.

Our results are limited by a lack of data on elements of the pan-TB regimen, in particular around the acquisition and effect of resistance. Those data are limited for current novel drugs such as bedaquiline and are based entirely on assumptions for other drugs that may compose future pan-TB regimens. We also approximated as zero the probability of acquiring resistance to multiple regimen components during 1 treatment course; however, there may be scenarios in which multiple drugs share resistance pathways and simultaneous acquisition is common. Further, our approach could be improved by the use of a transmission model, both for estimating the timescales of temporal trends and for estimating changes in absolute TB incidence as a result of more effective regimens. Our focus on cycles of transmission and proportions of patients with resistance could have led to an overestimate in the absolute prevalence of resistance generated by the pan-TB regimen, given that we did not fully account for reductions in transmission or potential disproportionate removal of DS-TB because of successful cure. Furthermore, our results are based on target regimen profiles, which assume that pan-TB regimens will be more effective than existing DS-TB regimens. We assume that if such regimens were available, they would be used at a minimum for patients with RR-TB, which leads to the counterintuitive standard-of-care scenario, where treatment outcomes for patients with RR-TB are better than for those with DS-TB.

Overall, we found that a pan-TB regimen adhering to the current target regimen profile is unlikely to drive an increase in complex resistance to both novel and older TB drugs. However, pan-TB regimens that are associated with both frequent acquisition of resistance and large associated reductions in efficacy, as well as pan-TB regimens that only marginally outperform the existing standard of care when introduced, could have short-lived viability as pan-TB regimens, requiring either regimen replacement or reintroduction of DST to maintain the improved health outcomes that a pan-TB regimen would initially offer. As new regimens are scaled up, TB programs will need to ensure effective support for adherence and regimen completion, implement systems to identify and effectively treat patients who experience treatment failure or TB recurrence and conduct ongoing surveillance to track any rise in resistance to the new regimens.

Appendix Additional information about potential of pan-tuberculosis treatment to drive emergence of novel resistance.
