# Supplementary material for: Potential of Pan-Tuberculosis Treatment to Drive Emergence of Novel Resistance
Source: Emerg Infect Dis. 2024 Aug;30(8):1571–9. doi: 10.3201/eid3008.240541 (PMC11286077; doi:10.3201/eid3008.240541)
Supplement: Appendix — Additional information about potential of pan-tuberculosis treatment to drive emergence of novel resistance. [file 24-0541-Techapp-s1.pdf]

Article DOI: https://doi.org/10.3201/eid3008.240541

EID cannot ensure accessibility for Appendix materials supplied by authors. Readers who have difficulty accessing Appendix content should contact the authors for assistance.

Potential of Pan-Tuberculosis Treatment to Drive Emergence of Novel Resistance

Appendix

Supplementary Methods

We estimated the proportion durably cured by different regimens in a separate piece of work (T.S. Ryckman et al, unpub. data, 2024), which followed a similar approach to that of Ryckman et al. (*1*) and is reproduced in brief here and available at GitHub (https://github.com/rycktessman/pan-tb-modeling), with parameter values shown in Appendix Table 2. This captured regimen assignment and initiation, pre-treatment losses to follow-up which varied by regimen assignment, and outcomes of treatment, and ultimately resulted in an estimation of the proportion of diagnosed patients who achieved durable microbiological cure. The probability of durable cure depended on regimen efficacy, duration, ease of adherence, forgiveness (i.e., the extent to which durable cure can occur despite missed doses), and resistance. Efficacy was defined as the proportion of patients curable by the regimen under optimal conditions of perfect adherence, retention in care, and complete initial regimen susceptibility; for the standard of care, efficacy estimates were based on clinical trial data (*2*,*3*). Starting from each regimen's efficacy, the probability of cure was adjusted downward for resistance to drugs in the regimen, early treatment discontinuation, and missed doses while on treatment. Discontinuation and adherence with standard of care regimens were based on programmatic data and control groups in trials of adherence-improving interventions, respectively. The impact of adherence varied by regimen forgiveness (*4*); for more forgiving regimens, more doses could be intermittently missed without affecting the probability of cure.

The hypothetical pan-TB regimen was informed by the WHO's minimal target regimen profile and ongoing regimen development efforts (*5*). In particular, the oral regimen was modeled as easier to adhere to, at least as forgiving, of shorter duration (3.5 months), and

as efficacious and safe as HRZE – and consisting of drugs with a lower population-wide prevalence of resistance than rifampin.

Durable cure and resistance acquisition rates depended on the resistance phenotype given different regimens (Appendix Table 3). When resistance to all drugs was present, the potential for cure by individualized regimens was parameterized based on longer regimens used for RR-TB before use of bedaquiline, pretomanid, or linezolid ("conventional second-line regimen"). When susceptibility to either B or X was retained (i.e., for RR/BR-TB or RR/XR-TB), the inclusion of that drug in an "X-based" or "B-based" individualized regimen would restore half of the incremental potential for cure that BX would offer for fully-susceptible TB. For a B-based individualized regimen (and similarly for an X-based regimen), the probability of acquiring resistance to B was estimated as the mean of the risk of acquisition of resistance during treatment of (i) drug susceptible TB with BX and (ii) XR-TB with BX.

Supplementary Results

Parameter values used for this illustration are the mean values we calculated previously (Appendix Table 1). For this set of parameter values, the pan-TB scenario resulted in fewer deaths, less treatment failure, and less drug resistance; however, in both scenarios, most TB that persisted after retreatment was DS-TB.

The likelihood of durable cure is higher under the pan-TB scenario irrespective of underlying prevalence of resistance in the population. This is a result of assumptions about the high rate of durable cure for the BX regimen, and the relatively minimal effect of existing resistance to regimen components on this. The likelihood of both increases as the prevalence of RR-TB increases (when RR-TB is likely to be undertreated in the standard of care scenario) and decreases as the prevalence of B resistance increases (when BR-TB is likely to be undertreated in the Pan-TB scenario).

References

1. Ryckman TS, Schumacher SG, Lienhardt C, Sweeney S, Dowdy DW, Mirzayev F, et al. Economic implications of novel regimens for tuberculosis treatment in three high-burden countries: a modelling analysis. Lancet Glob Health. 2024;12:e995–1004. PubMed https://doi.org/10.1016/S2214-109X(24)00088-3

2. Gegia M, Winters N, Benedetti A, van Soolingen D, Menzies D. Treatment of isoniazid-resistant tuberculosis with first-line drugs: a systematic review and meta-analysis. Lancet Infect Dis. 2017;17:223–34. PubMed https://doi.org/10.1016/S1473-3099(16)30407-8

3. Nyang'wa B-T, Berry C, Kazounis E, Motta I, Parpieva N, Tigay Z, et al.; TB-PRACTECAL Study Collaborators. A 24-week, all-oral regimen for rifampin-resistant tuberculosis. N Engl J Med. 2022;387:2331–43. PubMed https://doi.org/10.1056/NEJMoa2117166

4. Imperial MZ, Nahid P, Phillips PPJ, Davies GR, Fielding K, Hanna D, et al. A patient-level pooled analysis of treatment-shortening regimens for drug-susceptible pulmonary tuberculosis. Nat Med. 2018;24:1708–15. PubMed https://doi.org/10.1038/s41591-018-0224-2

5. World Health Organization. Target regimen profiles for tuberculosis treatment. 2023 Nov [cited 2023 Dec 7). https://www.who.int/publications-detail-redirect/9789240081512

6. World Health Organization. Global tuberculosis report 2023. 2023 [cited 2024 Apr 12]. https://www.who.int/teams/global-tuberculosis-programme/tb-reports/global-tuberculosis-report-2023

7. Cox H, Kebede Y, Allamuratova S, Ismailov G, Davletmuratova Z, Byrnes G, et al. Tuberculosis recurrence and mortality after successful treatment: impact of drug resistance. PLoS Med. 2006;3:e384. PubMed https://doi.org/10.1371/journal.pmed.0030384

8. He GX, Xie YG, Wang LX, Borgdorff MW, van der Werf MJ, Fan JH, et al. Follow-up of patients with multidrug resistant tuberculosis four years after standardized first-line drug treatment. PLoS One. 2010;5:e10799. PubMed https://doi.org/10.1371/journal.pone.0010799

9. Timm J, Bateson A, Solanki P, Paleckyte A, Witney AA, Rofael SAD, et al. Baseline and acquired resistance to bedaquiline, linezolid and pretomanid, and impact on treatment outcomes in four tuberculosis clinical trials containing pretomanid. PLOS Glob Public Health. 2023;3:e0002283. PubMed https://doi.org/10.1371/journal.pgph.0002283

10. Menzies D, Benedetti A, Paydar A, Martin I, Royce S, Pai M, et al. Effect of duration and intermittency of rifampin on tuberculosis treatment outcomes: a systematic review and meta-analysis. PLoS Med. 2009;6:e1000146. PubMed https://doi.org/10.1371/journal.pmed.1000146

11. Kendall EA, Azman AS, Cobelens FG, Dowdy DW. MDR-TB treatment as prevention: The projected population-level impact of expanded treatment for multidrug-resistant tuberculosis. PLoS One. 2017;12:e0172748. PubMed https://doi.org/10.1371/journal.pone.0172748

12. Perumal R, Bionghi N, Nimmo C, Letsoalo M, Cummings MJ, Hopson M, et al. Baseline and treatment-emergent bedaquiline resistance in drug-resistant tuberculosis: a systematic review

and meta-analysis. Eur Respir J. 2023;62:2300639. PubMed https://doi.org/10.1183/13993003.00639-2023

13. Ismail NA, Omar SV, Moultrie H, Bhyat Z, Conradie F, Enwerem M, et al. Assessment of epidemiological and genetic characteristics and clinical outcomes of resistance to bedaquiline in patients treated for rifampicin-resistant tuberculosis: a cross-sectional and longitudinal study. Lancet Infect Dis. 2022;22:496–506. PubMed https://doi.org/10.1016/S1473-3099(21)00470-9

14. Mallick JS, Nair P, Abbew ET, Van Deun A, Decroo T. Acquired bedaquiline resistance during the treatment of drug-resistant tuberculosis: a systematic review. JAC Antimicrob Resist. 2022;4:dlac029. **PMID: 35356403**

15. Lew W, Pai M, Oxlade O, Martin D, Menzies D. Initial drug resistance and tuberculosis treatment outcomes: systematic review and meta-analysis. Ann Intern Med. 2008;149:123–34. PubMed https://doi.org/10.7326/0003-4819-149-2-200807150-00008

16. Li S, Tan Y, Deng Y, Bai G, Huang M, Shang Y, et al. The emerging threat of fluroquinolone-, bedaquiline-, and linezolid-resistant *Mycobacterium tuberculosis* in China: Observations on surveillance data. J Infect Public Health. 2024;17:137–42. PubMed https://doi.org/10.1016/j.jiph.2023.11.018

17. Derendinger B, Dippenaar A, de Vos M, Huo S, Alberts R, Tadokera R, et al. Bedaquiline resistance in patients with drug-resistant tuberculosis in Cape Town, South Africa: a retrospective longitudinal cohort study. Lancet Microbe. 2023;4:e972–82. PubMed https://doi.org/10.1016/S2666-5247(23)00172-6

18. Ismail NA, Aono A, Borroni E, Cirillo DM, Desmaretz C, Hasan R, et al. A multimethod, multicountry evaluation of breakpoints for bedaquiline resistance determination. Antimicrob Agents Chemother. 2020;64:e00479–20. PubMed https://doi.org/10.1128/AAC.00479-20

19. Liu Y, Gao M, Du J, Wang L, Gao J, Shu W, et al. Reduced susceptibility of *Mycobacterium tuberculosis* to bedaquiline during antituberculosis treatment and its correlation with clinical outcomes in China. Clin Infect Dis. 2021;73:e3391–7. PubMed https://doi.org/10.1093/cid/ciaa1002

20. Subbaraman R, Nathavitharana RR, Satyanarayana S, Pai M, Thomas BE, Chadha VK, et al. The tuberculosis cascade of care in India’s public sector: a systematic review and meta-analysis. PLoS Med. 2016;13:e1002149. PubMed https://doi.org/10.1371/journal.pmed.1002149

21. Naidoo P, Theron G, Rangaka MX, Chihota VN, Vaughan L, Brey ZO, et al. The South African tuberculosis care cascade: estimated losses and methodological challenges. J Infect Dis. 2017;216(suppl_7):S702–13. PubMed https://doi.org/10.1093/infdis/jix335

22. Cox H, Dickson-Hall L, Ndjeka N, Van't Hoog A, Grant A, Cobelens F, et al. Delays and loss to follow-up before treatment of drug-resistant tuberculosis following implementation of Xpert MTB/RIF in South Africa: A retrospective cohort study. PLoS Med. 2017;14:e1002238. PubMed https://doi.org/10.1371/journal.pmed.1002238

23. Kruk ME, Schwalbe NR, Aguiar CA. Timing of default from tuberculosis treatment: a systematic review. Trop Med Int Health. 2008;13:703–12. PubMed https://doi.org/10.1111/j.1365-3156.2008.02042.x

24. Collaborative Group for the Meta-Analysis of Individual Patient Data in MDR-TB treatment–2017, Ahmad N, Ahuja SD, Akkerman OW, Alffenaar JWC, Anderson LF, et al. Treatment correlates of successful outcomes in pulmonary multidrug-resistant tuberculosis: an individual patient data meta-analysis. Lancet. 2018;392:821–34. **PMID: 30215381**

25. World Health Organization. Consolidated guidelines on tuberculosis: drug-susceptible tuberculosis treatment. 2022 [cited 2023 Mar 16]. https://www.who.int/publications-detail-redirect/9789240048126

26. Stagg HR, Lewis JJ, Liu X, Huan S, Jiang S, Chin DP, et al. Temporal factors and missed doses of tuberculosis treatment. A causal associations approach to analyses of digital adherence data. Ann Am Thorac Soc. 2020;17:438–49. PubMed https://doi.org/10.1513/AnnalsATS.201905-394OC

27. Maraba N, Orrell C, Chetty-Makkan CM, Velen K, Mukora R, Page-Shipp L, et al. Evaluation of adherence monitoring system using evriMED with a differentiated response compared to standard of care among drug-sensitive TB patients in three provinces in South Africa: a protocol for a cluster randomised control trial. Trials. 2021;22:389. PubMed https://doi.org/10.1186/s13063-021-05337-y

28. Liu X, Thompson J, Dong H, Sweeney S, Li X, Yuan Y, et al. Digital adherence technologies to improve tuberculosis treatment outcomes in China: a cluster-randomised superiority trial. Lancet Glob Health. 2023;11:e693–703. PubMed https://doi.org/10.1016/S2214-109X(23)00068-2

Appendix Table 1. Parameter values and sources.

Parameter	Description	Value*	Source
Regimen effectiveness			
E_R	Proportion of DS-TB durably cured by rifamycin-based regimen	70.9% [58.5- 79.3%]	(T.S. Ryckman et al, unpub. data, 2024)
E_{BX}	Proportion of DS-TB or RR-TB durably cured by pan-TB regimen	76.3% [68.8- 83.1%]	(T.S. Ryckman et al, unpub. data, 2024), based on a 2-mo pan-TB regimen
E_{ind}	Proportion of TB durably cured by individualized regimen	43.9% [33.7- 53.6%]	(T.S. Ryckman et al, unpub. data, 2024)
CFR	Proportion of poor outcomes (i.e., no durable cure) that result in death	48.3% [40.3- 52.5%]	(*6*), uncertainty taken from regional variation
Impact of resistance on cure			
P_R	Risk ratio of cure for rifamycin-based regimen given initial RR-TB	0.35 [0.22- 0.5]	(T.S. Ryckman et al, unpub. data, 2024) (*7*,*8*)
P_B	Risk ratio of cure for pan-TB regimen given initial BR-TB	0.75 [0.54- 0.91]	(T.S. Ryckman et al, unpub. data, 2024) (*9*)
P_X	Risk ratio of cure for pan-TB regimen given initial XR-TB	0.75 [0.54- 0.91]	Assumed to be similar to P_B
P_{BX}	Risk ratio of cure for pan-TB regimen given BR- and XR-TB	0.35 [0.22- 0.5]	Assumed to be similar to P_R
Resistance acquisition			
S_R	Probability of acquired resistance to R after rifamycin-based treatment (if initially RS-TB)	0.6% [0.3- 1.2%]	(*10*,*11*)
S_B	Probability of acquired resistance to B after pan-TB treatment (if initially BS and XS)	1% [0.3%–2.3%]	(*9*,*12*–*14*), based on observational studies and trial results; point estimate reflects where these two intersect
S_X	Probability of acquired resistance to X after pan-TB treatment (if initially BS and XS)	1% [0.3%–2.3%]	Assumed to be similar to S_B, based on (*2*)
Q	Risk ratio for B or X resistance acquisition given pre-existing X or B resistance, respectively	7.5 [4.0- 16.0]	Assumption based on (*2*,*15*) for other drugs
Drug susceptibility testing			
R_soc_{new}	R DST for new patients, standard of care scenario	44.10% [33.1%–55.1%]	(*6*) weighted by the proportion with bacteriological confirmation with an assumed 25% uncertainty interval
BX_soc_{new}	B and X DST coverage for new patients with known RR-TB, standard of care scenario	49.0% [36.8%–61.3%]	(*6*) using fluoroquinolone testing coverage with an assumed 25% uncertainty interval
R_soc_{retR}	R DST coverage for retreatment patients previously treated with R, standard of care scenario	80.0% [60.0%–100%]	Assumed higher than R_soc_{new}
R_soc_{retBX}	R DST coverage for retreatment patients previously treated with BX, standard of care scenario	100%	Assumption
BX_soc_{retR}	B and X DST coverage for retreatment patients previously treated with R with known RR-TB, standard of care scenario	49.0% [36.8%–61.3%]	Assumed to be similar to new patients in the standard of care scenario
BX_soc_{retBX}	B and X DST coverage for retreatment patients previously treated with BX with known RR-TB, standard of care scenario	60.0% [45.0%–75.0%]	Assumption, higher than retreatment patients in the standard of care scenario

Parameter	Description	Value*	Source
R_pan	R DST coverage for retreatment patients, pan-TB scenario	44.1% [33.1%–55.1%]	Assumed to be similar to new patients in the standard of care scenario
BX_pan	B and X DST coverage for retreatment patients with known RR-TB, pan-TB scenario	0% or 49.0% [36.8%–61.3%]	Assumed to be similar to new patients in the standard of care scenario
Baseline prevalence of resistance			
$prev_{DS}$	Initial prevalence of DS-TB	95.70%	(*6*)
$prev_{RR}$	Initial prevalence of RR-TB	4.20%	(*6*) weighting new and previously treated patients
$prev_{BR}$	Initial prevalence of BR-TB	0.20%	(*9*,*16*)
$prev_{XR}$	Initial prevalence of XR-TB	0.00%	Assumption
$prev_{RRBR}$	Initial prevalence of RR/BR-TB	0.09%	(*12*,*17*–*19*)
$prev_{RRXR}$	Initial prevalence of RR/XR-TB	0.00%	Assumption
$prev_{BRXR}$	Initial prevalence of BR/XR-TB	0.00%	Assumption
$prev_{RRBRXR}$	Initial prevalence of RR/BR/XR-TB	0.00%	Assumption

*All parameters were assumed to follow β distribution (fitted to the median and 95% uncertainty interval shown) except for the parameter Q, which followed a uniform distribution.
DS-TB = drug susceptible tuberculosis, RR-TB = rifampin-resistant tuberculosis, BR-TB = diarylquinoline resistant tuberculosis, XR-TB = tuberculosis resistant to additional novel drug X.

Appendix Table 2. Parameters used to estimate durable cure rates shown in Appendix Table 1.

Parameter	Regimen	Estimate [95% uncertainty interval]	Distribution used for parameter sampling*	Sources/Notes
Pre-treatment LTFU if assigned to RS SOC or Pan-TB regimen	–	13% [8-19%]	Normal (mean 0.134, sdev 0.028)	Subbaraman et al. 2016 (*20*); Naidoo et al. 2017 (*21*)
Additional pre-treatment LTFU if assigned to a separate care pathway (RR SOC or individualized regimen)	–	16% [7-27%]	Beta (8, 42)	Based on Subbaraman et al. 2016 (*20*), Cox et al. 2017 (*22*), and WHO notifications data (*6*)
Weekly probability of early discontinuation	India	0.13% [0.08-0.18%]	Beta (26, 19923)	WHO data from a range of countries (*6*) Assumed to be constant over time based on Kruk et al. 2008 (*23*)
	South Africa	0.41% [0.31-0.52%]	Beta (58, 14168)	
	Philippines	0.14% [0.08-0.20%]	Beta (20, 14824)	
Efficacy	RS-TB SOC	95% [93-97%]		Gegia et al. 2017 (*2*)
	Pan-TB TRP	95% [93-97%]	Same as RS SOC, not modeled independently	
	Individualized	75% [67-83%]	Beta (75, 25)	Based on outcomes of MDR-TB patients pre-BPaL/BPaLM (*24*)
Duration	RS-TB SOC	24 weeks	NA – no uncertainty in duration was modeled	WHO guidelines (*25*)
	Pan-TB	14 weeks		WHO target regimen profile (minimal target) (*5*)
	Individualized	18 months		Based on RR regimens pre-BPaL/BPaLM
% patients w/ < 70% adherence	RS-TB SOC	38% [28-48%]	Multinomial (100, 0.379)	Median of control groups in 3 adherence-improving intervention studies (*26*–*28*)
	Pan-TB	14% [12-16%]	Multinomial (100, 0.14)	Best of control groups in 3 adherence-improving 3 intervention studies (*26*–*28*), based on minimal TRP target of better tolerability than the standard of care (*5*)
	Individualized	38% [28-48%]	Same as RR-TB	Assumed same as BPaL/BPaLM

Parameter	Regimen	Estimate [95% uncertainty interval]	Distribution used for parameter sampling*	Sources/Notes
% patients w/ 70-90% adherence	RS-TB SOC	31% [18-46%]	Multinomial (100, 0.312)	Median of control groups in 3 adherence-improving intervention studies (*26*–*28*)
	Pan-TB	34% [31-38%]	Multinomial (100, 0.35)	Best of control groups in 3 adherence-improving 3 intervention studies (*26*–*28*), based on minimal TRP target of better tolerability than the standard of care (*5*)
	Individualized	31% [18-46%]	Same as RR-TB	Assumed same as HRZE.
% patients w/ ≥ 90% adherence	RS-TB SOC	31% [22-40%]	Multinomial (100, 0.309)	Median of control groups in 3 adherence-improving intervention studies (*26*–*28*)
	Pan-TB	51% [47-55%]	Multinomial (100, 0.51)	Best of intervention groups in 3 adherence-improving 3 intervention studies (*26*–*28*), based on minimal TRP target of better tolerability than the standard of care (*5*)
	Individualized	31% [22-40%]	Same as RR-TB	Assumed same as HRZE.
Forgiveness (nonadherence threshold above which probability of cure < efficacy)	RS-TB SOC	10%	NA – no uncertainty was modeled in the forgiveness thresholds, but uncertainty was included in the relative probability of cure above vs. below the forgiveness threshold (next row).	Imperial et al. (*4*)
	Pan-TB oral	15%		Value from WHO minimal TRP (*5*)
	Individualized	10%		Assumed same as HRZE.
Relative probability of cure if missed doses exceed the forgiveness threshold	All	82% [63-94%]	Simulated using the confidence intervals from Imperial et al. (*4*), assuming Wald distributions.	Imperial et al. (*4*)

"RS" = rifampin-susceptible; "SOC" = standard of care; "HRZE" = 6 months of isoniazid, rifampin, pyrazinamide, and ethambutol (the standard of care regimen for RS-TB); "BPaLM" = 6 months of bedaquiline, pretomanid, linezolid, and moxifloxacin (the standard of care regimen for RR-TB). "TRP" = target regimen profile.
Both point estimates/means and uncertainty intervals have been estimated from the sources indicated in the "Sources/Notes" column, unless otherwise noted.
*For the uncertainty distributions (column 4), the normal distribution is displayed with mean and standard deviation in parentheses, the beta distribution is displayed with alpha and beta in parentheses, where the mean of a beta distribution is equal to alpha divided by the sum of alpha and beta, the multinomial distribution is displayed with size and probability parameters in parentheses, and the gamma distribution is displayed with shape and scale parameters in parentheses.

Appendix Table 3. Durable cure and acquisition of resistance for different resistance phenotypes given different regimens (assuming mean parameter values from Appendix Table 1).

		Resistance type							
Outcome	Regimen	DS-TB	RR-TB	BR-TB	XR-TB	RR+ BR-TB	RR+ XR-TB	BR+ XR-TB	RR+BR+ XR-TB
Durable cure	R	E_R = 70.9%	$E_R \cdot P_R$ = 24.8%	E_R = 70.9%	E_R = 70.9%	$E_R \cdot P_R$ = 24.8%	$E_R \cdot P_R$ = 24.8%	E_R = 70.9%	$E_R \cdot P_R$ = 24.8%
	BX	E_{BX} = 79.7%	E_{BX} = 79.7%	$E_{BX} \cdot P_B$ = 59.8%	$E_{BX} \cdot P_X$ = 59.8%	$E_{BX} \cdot P_B$ = 59.8%	$E_{BX} \cdot P_X$ = 59.8%	$E_{BX} \cdot P_{BX}$ = 27.9%	$E_{BX} \cdot P_{BX}$ = 27.9%
	B-based	–	–	–	–	–	69.70%	–	–
	X-based	–	–	–	–	69.70%	–	–	–
	Conv 2nd- line	–	–	–	–	–	–	–	E_{ind} = 43.9%
RR Acquisition	R	S_R = 0.6%	–	S_R = 0.6%	S_R = 0.6%	–	–	S_R = 0.6%	–
BR Acquisition	BX	S_B = 1.0%	S_B = 1.0%	–	$S_B \cdot Q$ = 7.5%	–	$S_B \cdot Q$ = 7.5%	–	–
	B- based	–	–	–	–	–	4.30%	–	–
XR Acquisition	BX	S_X = 1.0%	S_X = 1.0%	$S_X \cdot Q$ = 7.5%	–	$S_X \cdot Q$ = 7.5%	–	–	–
	X- based	–	–	–	–	4.30%	–	–	–

*TB = tuberculosis, DS-TB = drug-susceptible tuberculosis, RR-TB = rifampin-resistant tuberculosis, BR-TB = diarylquinoline resistant tuberculosis, XR-TB = tuberculosis resistant to additional novel drug X, ER = proportion of DS-TB durably cured by rifamycin-based regimen, E_{BX} = proportion of DS-TB durably cured by pan-TB regimen, E_{ind} = proportion of TB durably cured by individualized regimen, P_R = Risk ratio of cure for rifamycin-based regimen given initial RR-TB, P_B = Risk ratio of cure for pan-TB regimen given initial BR-TB, P_X = Risk ratio of cure for pan-TB regimen given initial XR-TB, P_{BX} = Risk ratio of cure for pan-TB regimen given BR- and XR-TB, S_R = Probability of acquired RR-TB after rifamycin-based treatment (if initially RS-TB), S_B = Probability of acquired BR-TB after pan-TB treatment (if initially BS and XS), S_X = Probability of acquired XR-TB after pan-TB treatment (if initially BS and XS), Q = Risk ratio for B or X resistance acquisition given pre-existing X or B resistance, respectively.

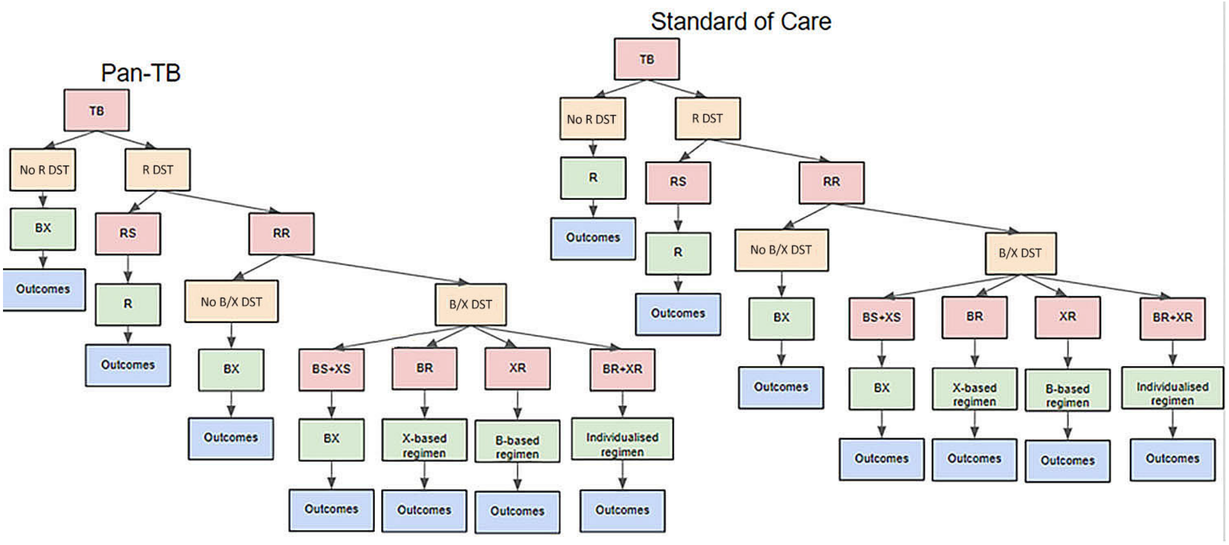

Appendix Figure 1. Treatment pathways for previously treated patients, comparing the Pan-TB scenario (left) with standard of care (right). TB = tuberculosis, RS-TB = rifampin-susceptible tuberculosis, RR-TB = rifampin-resistant tuberculosis, BR-TB = diarylquinoline resistant tuberculosis, XR-TB = tuberculosis resistant to additional novel drug X, R DST = rifampin drug susceptibility testing, B/X DST = diarylquinoline and other novel drug(s) susceptibility testing, R = rifampin-based regimen, BX = pan-TB regimen.

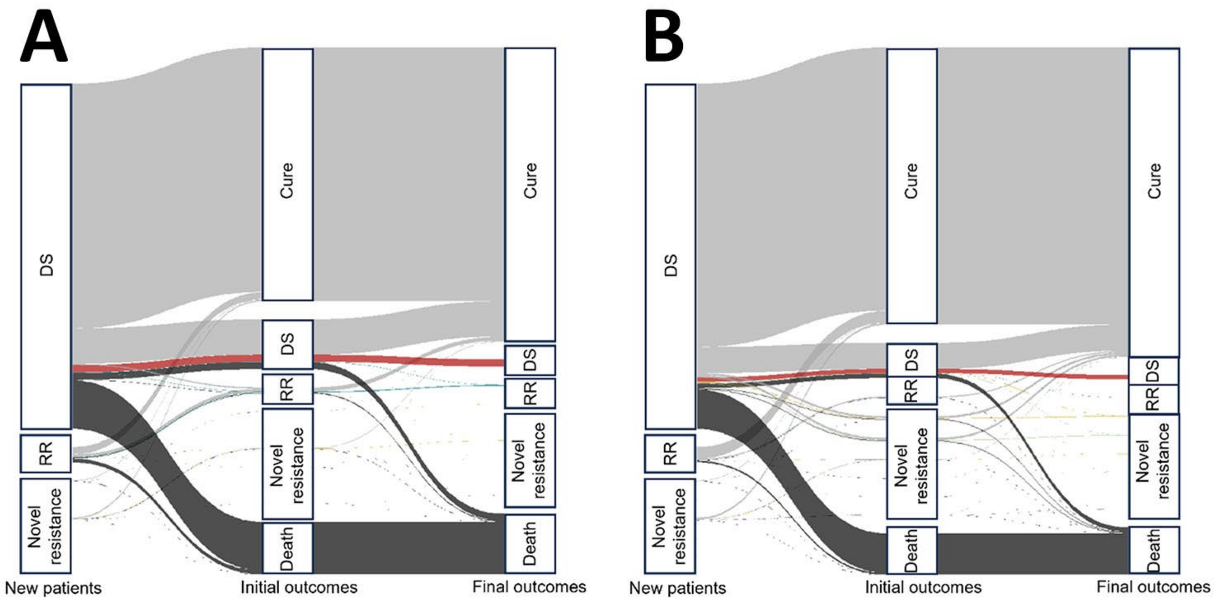

Appendix Figure 2. Sankey diagram using mean parameter values for the (a) standard of care and (b) pan-TB scenario. Colors indicate the final treatment outcome. DS = drug susceptible tuberculosis, RR = rifampin-resistant tuberculosis, Novel resistance = tuberculosis resistant to a diarylquinoline and/or novel drug X.

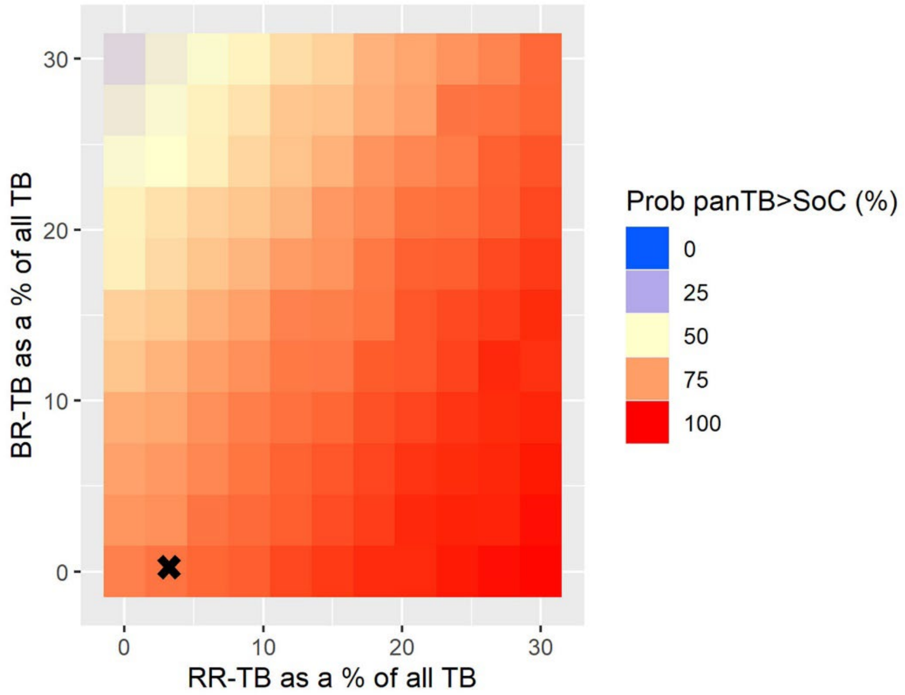

Appendix Figure 3. Probability that pan-TB leads to a higher durable cure rate than the standard of care after 1 cohort of patients for varying initial prevalence of resistance. Red indicates where pan-TB TB performs better, blue where SoC performs better. Both RR-TB and BR-TB are varied as a proportion of all TB, where RR+BR-TB is the product of both. No other forms of resistance are initially present. X indicates current estimated prevalence of resistance globally.

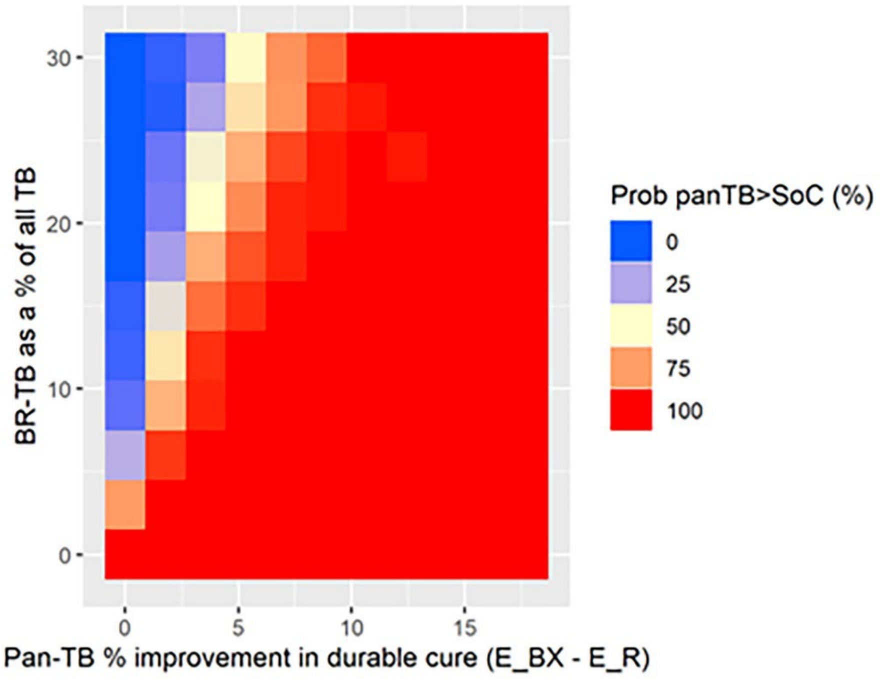

Appendix Figure 4. Effect of difference in cure rates on relative performance of regimens for varying prevalence of B resistance.

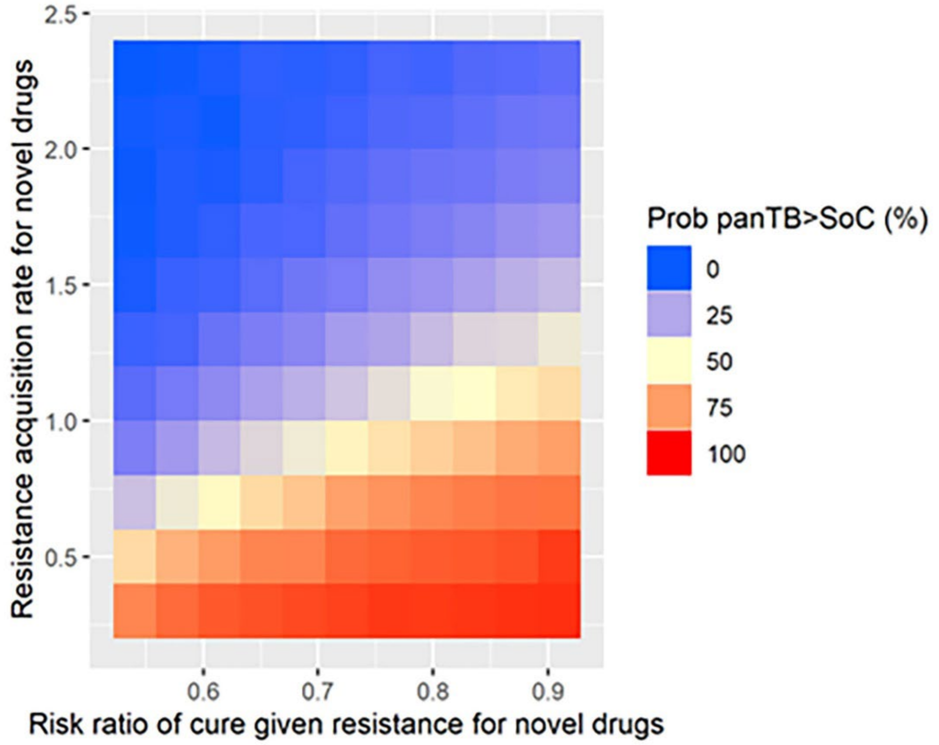

Appendix Figure 5. Probability that the pan-TB scenario leads to higher durable cure compared to the standard of care after 10 cohorts. Red indicates where pan-TB TB performs better, blue where SoC performs better. Note parameters values (resistance acquisition rate and risk ratio of cure) are varied for both novel drug types simultaneously.

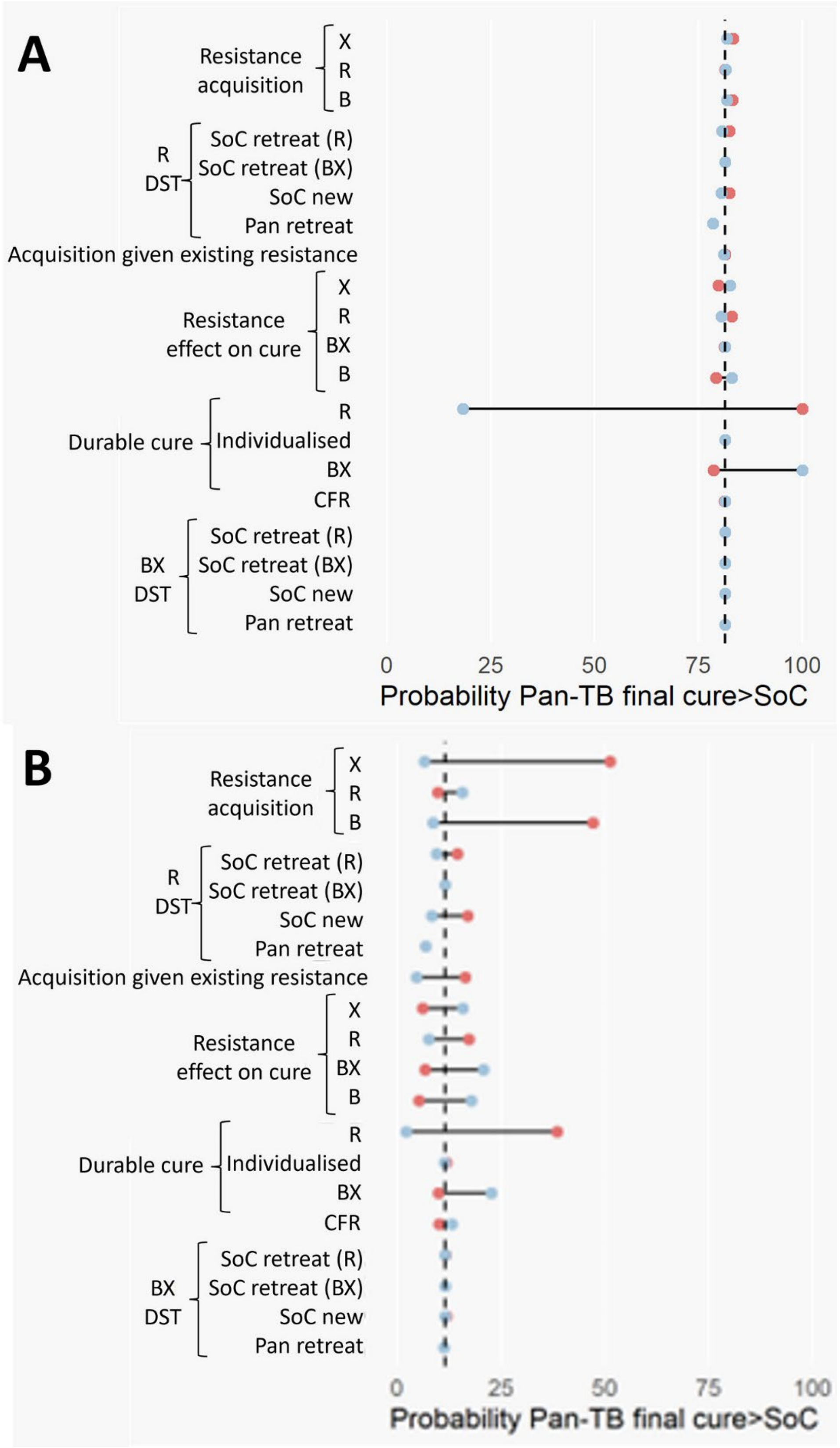

Appendix Figure 6. Univariate sensitivity analysis, sampling a parameter set and fixing each parameter in turn at the extremes of its 95% uncertainty interval, where the mean estimate and upper bound of the uncertainty interval for acquisition of resistance to novel drugs have been increased. We based these new estimates on high rates of resistance acquisition seen under programmatic

conditions (e.g., in centers that were not green-light approved), such that the probability of acquired resistance to B after pan-TB treatment (if initially BS and XS) = 2.3% [0.3%–8%], and the probability of acquired resistance to X after pan-TB treatment (if initially BS and XS) = 1% [0.3%–8%]. Comparing likelihood over that durable cure in the pan-TB scenario is greater than the standard of care scenario after (a) one cohort of treatment (b) ten cohorts. Blue circles represent low parameter values, red circles high parameter values.

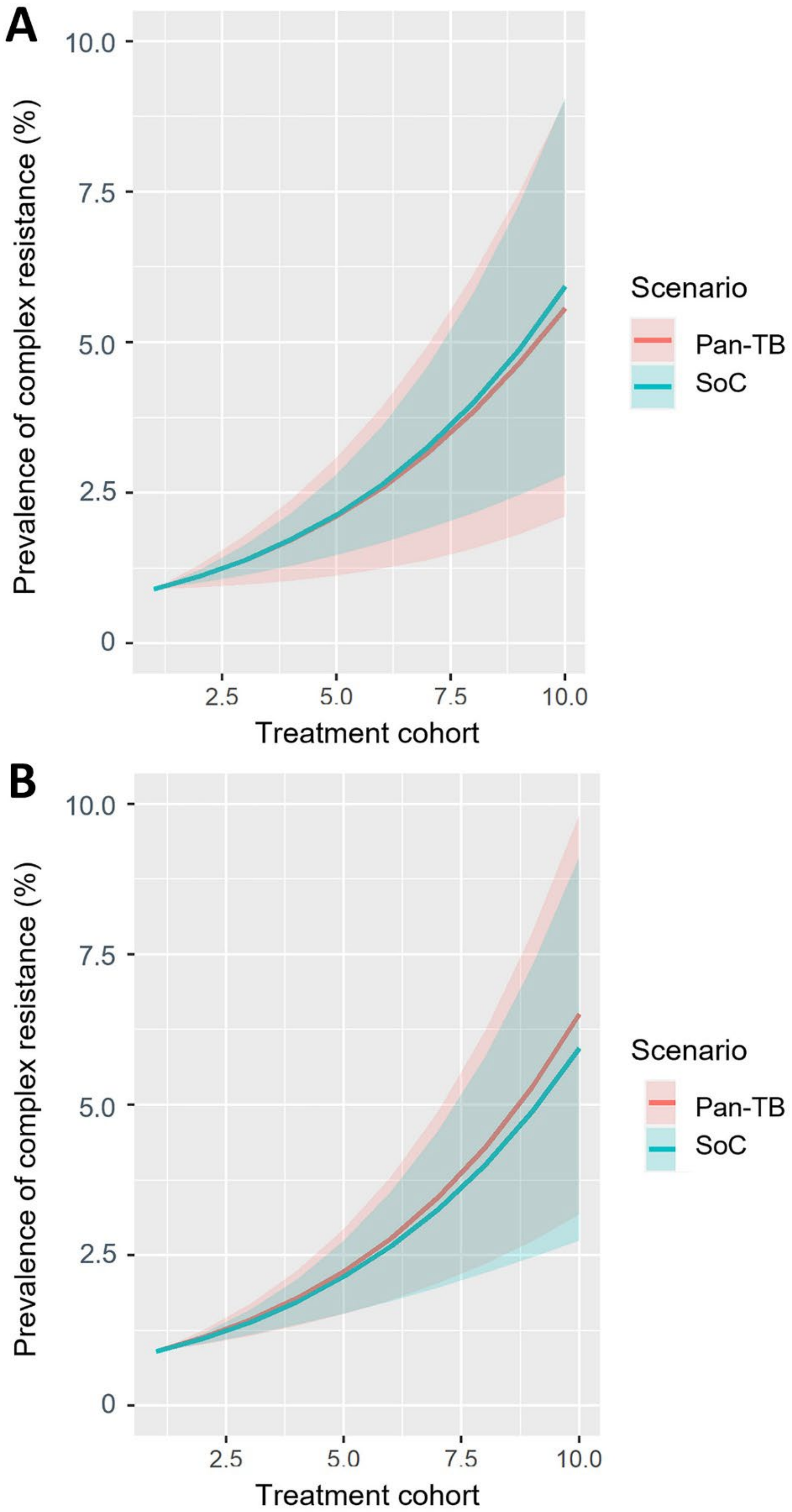

Appendix Figure 7. Prevalence of “complex” resistance to both R and either B and/or X over multiple cohorts where (a) B/X DST availability for retreatment patients with known RR-TB is zero, or (b) R DST availability for retreatment patients is 100%. Shaded areas indicate 95% uncertainty intervals. DS-TB = drug susceptible tuberculosis, RR-TB = rifampin-resistant tuberculosis, BR-TB = diarylquinoline resistant tuberculosis, XR-TB = tuberculosis resistant to novel drug X.

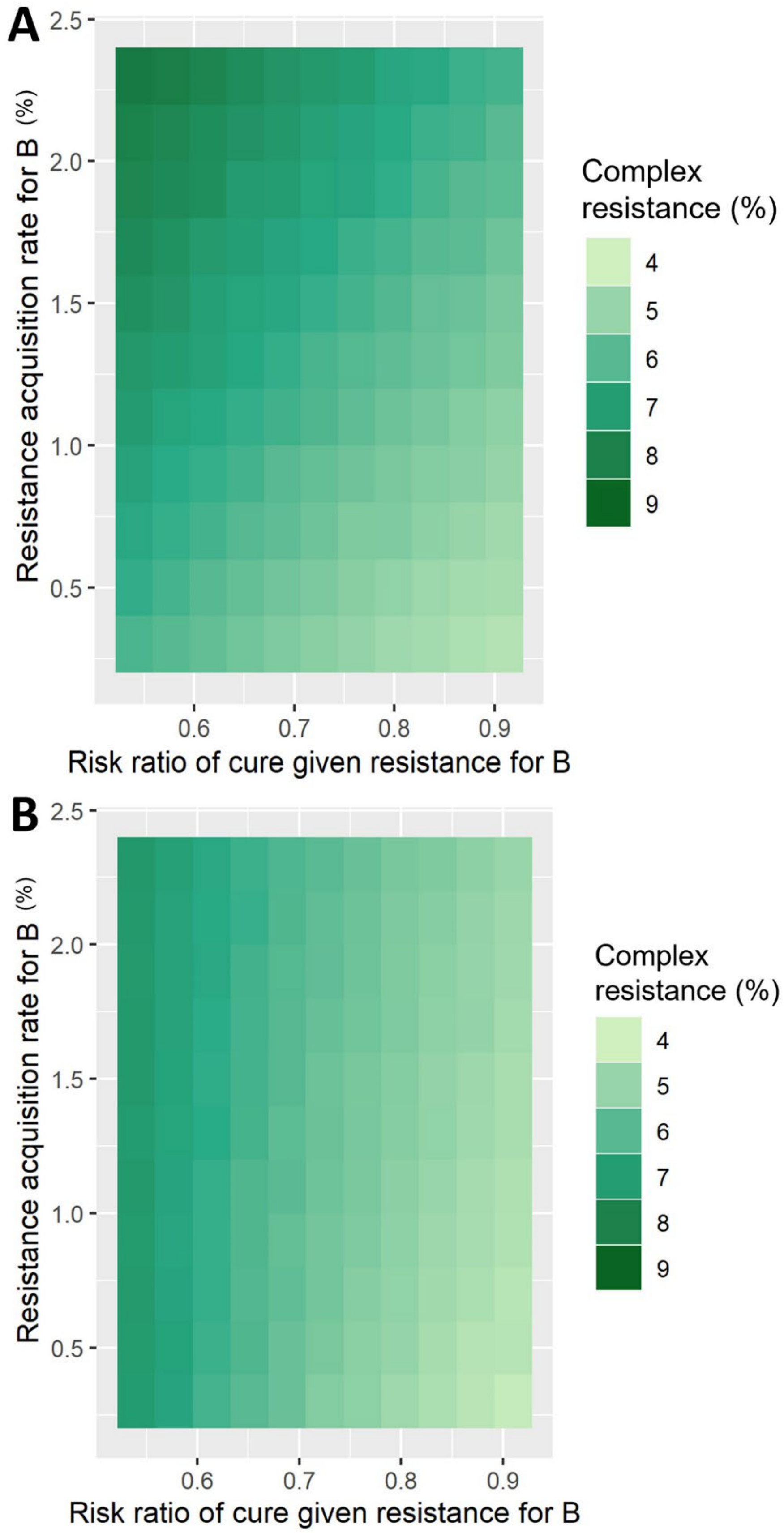

Appendix Figure 8. Prevalence of "complex" resistance both to R and to either B and/or X after 10 cohorts for (a) standard of care and (b) pan-TB.
